# Comparison of Yen, Beta, Wits, K angles with ANB in assessing sagittal dysplasia: A cephalometric study

**DOI:** 10.6026/973206300214275

**Published:** 2025-12-15

**Authors:** Rameshwar Arun Munde, Amit Handa, Dipali Raghunath Mane, Akansha Katkar, Nikita Bhagwan, Himanshu Shrivastava, Aditi Sarda

**Affiliations:** 1 Department of Orthodontics and Dentofacial Orthopaedics, Aditya Dental College, Beed, Maharashtra, India; 2 Department of Conservative Dentistry and Endodontics, Aditya Dental College, Beed, Maharashtra, India; 3 Department of Oral Pathology and Microbiology, Aditya Dental College, Beed, Maharashtra, India

**Keywords:** ANB angle, Yen angle, Beta angle, Wits appraisal, K angle, sagittal skeletal dysplasia

## Abstract

Accurate assessment of sagittal skeletal discrepancies is crucial in orthodontic diagnosis. The commonly used ANB angle has limitations influenced by craniofacial growth and landmark variability. This study compared Yen angle, Beta angle, Wits appraisal and K angle with ANB angle in 120 cephalograms of adults aged 18-30 years. Statistical analysis showed Yen and Beta angles had the strongest correlation and predictive value, followed by K angle, while Wits appraisal was less reliable. Hence, Yen and Beta angles serve as more dependable alternatives to ANB angle in diagnosing sagittal dysplasia.

## Background:

The major goals of orthodontic treatment are to achieve functional efficiency, structural balance and aesthetic harmony. Developing an effective treatment plan requires accurate diagnosis, which is based on a combination of clinical examinations, model analysis and radiographic imaging, including orthopantomograms, lateral cephalograms and posteroanterior cephalograms [1]. Among these, cephalometric analysis remains the most valuable tool for assessing skeletal and dental relationships in the sagittal, vertical and transverse planes [2]. Malocclusions are typically analyzed in three dimensions: transverse, anteroposterior and vertical. Of these, anteroposterior discrepancies are of particular concern, as they directly influence both function and facial esthetics, making their assessment critical in orthodontics [3]. Correct evaluation of sagittal jaw relationships is essential for classifying skeletal patterns, guiding treatment planning and predicting outcomes. Several cephalometric indicators have been introduced to assess these relationships, including the ANB angle, Wits appraisal, Beta angle, Yen angle and K angle, each with distinct strengths and inherent limitations [4]. The ANB angle has long been considered the gold standard for evaluating anteroposterior discrepancies. However, it is influenced by numerous factors, including age, growth-related jaw rotations, vertical jaw development and the length and position of the anterior cranial base [5]. Additionally, difficulty in consistently locating cephalometric landmarks such as point A may further compromise its reliability [6]. To overcome these limitations, Jacobson proposed the Wits appraisal, which measures sagittal discrepancies independently of cranial landmarks by referencing the occlusal plane. While this method reduces dependence on point N, it introduces variability since the occlusal plane itself may change with growth or treatment [7]. The Yen angle was developed as another alternative, designed to reduce the influence of growth and allow application even during mixed dentition. Despite these advantages, jaw rotation may still obscure true sagittal relationships [8]. The Beta angle, introduced in 2004, assesses the severity of skeletal dysplasia with minimal effect from jaw rotations. Nonetheless, like ANB, it uses point A, which can be altered during orthodontic treatment [9]. More recently, the K angle has emerged as a consistent diagnostic tool, reported to maintain reliability across skeletal classes, age groups and genders. Given these considerations, identifying the most reliable parameter for diagnosing sagittal skeletal discrepancies remains clinically significant [10]. Therefore, this study is essential to determining which cephalometric measurement-Yen angle, Beta angle, Wits appraisal, or K angle-serves as the most dependable alternative to the ANB angle in routine orthodontic practice.

## Methodology:

This study was conducted in the Department of Orthodontics and Dentofacial Orthopedics. Standardized pretreatment lateral cephalograms of 120 orthodontic patients were collected from the Department of Oral Medicine and Radiology. The inclusion criteria consisted of patients with permanent dentition and no missing teeth, no previous history of orthodontic treatment, an age range of 18 to 30 years and cephalograms of high clarity. The exclusion criteria included patients with a history of orthodontic treatment, edentulous spaces, a history of trauma, congenital deformities, marked asymmetry, and poor-quality radiographs. Standardized pretreatment lateral cephalograms of randomly selected individuals reporting to the Department of Orthodontics and Dentofacial Orthopedics in the college were considered. A total of 120 individuals were selected and their cephalograms were recorded and printed on X-ray printing sheets. These lateral cephalograms were traced on tracing paper using a 0.5 mm HB drawing pencil and tracing table. Tracings were performed for five parameters, namely ANB angle, Yen angle, Beta angle, K angle and Wits appraisal.

Each parameter was categorized into skeletal Class I, II, or III according to their respective assessment methods and the other four parameters were compared with the ANB angle. The ANB angle was defined as the angle between the N-A line and the N-B line at point N, with mean values ranging from 1° to 4° for Class I, greater than 4° for Class II and less than 1° for Class III. The Yen angle was measured between the S-M line and the M-G line at point M, with skeletal Class I values ranging from 117° to 123°, less than 117° for Class II and greater than 123° for Class III. The Beta angle was formed between the A-B line and the line from point A drawn perpendicular to the C-B line at point A, with mean values of 28° to 35° for Class I, less than 28° for Class II and greater than 35° for Class III. Wits appraisal was performed by projecting perpendiculars from point A and point B onto the occlusal plane, with the points of contact labeled AO and BO respectively. The AO-BO distance was considered, with a mean value of 0 mm in females and -1 mm in males for Class I, a positive reading (BO behind AO) for Class II and a negative reading (BO ahead of AO) for Class III. The K angle was measured using landmarks M (midpoint of the anterior maxilla), G (center of the mandibular symphysis) and C (apparent axis of the condyle). The K angle was defined as the angle between the M-G line and the line from point M perpendicular to the G-C line, with mean values ranging from 40° to 46° for skeletal Class I, less than 40° for Class II and greater than 46° for Class III. Data entry was performed using Microsoft Office Excel 2019 and statistical analysis was conducted with the Statistical Package for the Social Sciences (SPSS), version 22. Descriptive statistics, including mean and standard deviation, were calculated for continuous variables, while frequencies and percentages were used for categorical variables. The significance level was set at p = 0.05 for statistical significance and p = 0.001 for high statistical significance. Data normality was assessed using the Shapiro-Wilk test. Pearson correlation analysis was applied to examine the relationship between different skeletal classes and regression analysis was performed to evaluate the dependency between the parameters.

## Results:

Table 1 (see PDF) presents the comparative descriptive statistics of the five cephalometric parameters: ANB angle, Yen angle, Beta angle, Wits appraisal and K angle. The mean ANB angle was 3.58°, with a standard deviation (SD) of 3.07. The Yen angle had a mean of 120.58° and a standard deviation of 6.49. The Beta angle showed a mean of 29.44° and a slightly higher variability (SD = 6.91). The Wits appraisal had the lowest mean value of 1.94 mm and a standard deviation of 2.94. The K angle showed a mean of 40.65°, with a standard deviation of 5.04. Table 2 (see PDF) presents the Pearson correlation coefficients among five cephalometric parameters-ANB, Yen, Beta, Wits and K Angles-based on a sample of 120 subjects. Correlation values range from -1 to +1, where positive values indicate a direct relationship and negative values indicate an inverse relationship. All correlations in the table are statistically significant (p < 0.001), indicating that the observed relationships are unlikely to have occurred by chance. ANB Angle shows a strong negative correlation with the Yen angle (r = -0.70) and Beta angle (r = -0.66), suggesting that as ANB increases, Yen and Beta angles tend to decrease. Then K angle (r = -0.61) correlated, then moderately positively correlated with Wits appraisal (r = 0.55). Yen Angle demonstrates a strong positive correlation with K angle (r = 0.71) and a moderate positive correlation with the Beta angle (r = 0.57). It is negatively correlated with Wits appraisal (r = -0.31). Beta Angle is strongly positively correlated with K angle (r = 0.79) and moderately negatively correlated with Wits (r = -0.37). Wits Appraisal shows moderate positive correlation with ANB (r = 0.55) and weak to moderate negative correlations with the other parameters-Yen (r = -0.31), Beta (r = -0.37) and K (r = -0.32). K Angle is strongly positively correlated with Beta (r = 0.79) and Yen (r = 0.71), while being moderately negatively correlated with ANB (r = -0.61) and Wits (r = -0.32). Table 3 (see PDF) presents the Coefficient of Determination (R^2^) values, which indicate how well each cephalometric parameter explains the variation in the ANB angle. The Yen angle shows the highest R^2^ value of 0.54, meaning it explains 54% of the variance in ANB, making it the most predictive. This is followed by the Beta angle at 0.52, the K angle at 0.44 and the Wits appraisal at 0.37, which has the lowest predictive value. All parameters demonstrate a moderate to strong relationship with ANB, with Yen and Beta angles showing the strongest dependency.

## Discussion:

Accurate evaluation of sagittal jaw relationships is essential in orthodontic diagnosis and treatment planning. While malocclusions are assessed in transverse, vertical and sagittal planes, sagittal discrepancies are most concerning due to their impact on facial esthetics and function. The ANB angle has long been the gold standard for assessing sagittal skeletal relationships, but it is limited by sensitivity to Nasion position, jaw rotations and cranial base length. To overcome these drawbacks, alternative indicators such as the Yen angle, Beta angle, Wits appraisal and K angle have been developed, each relying on different landmarks and planes. This study compared these four parameters with the ANB angle to evaluate their reliability and accuracy, aiming to identify the most effective tools for clinical orthodontic assessment. Descriptive statistics were used to summarize and interpret the overall trends in the cephalometric measurements. The study shows that the mean and standard deviation (SD) for the ANB angle in our study were 3.58 ± 3.07, which closely aligns with the findings of Soni *et al.* [11]. Similarly, the Beta angle had a mean value of 29.44 ± 6.91, comparable to the results reported by Kapadia *et al.* [12]. Additionally, the mean and SD for yen angle (value: 120.58 ± 6.49) were nearly consistent with those reported by Neela *et al.* [13], who observed values of 119.79 ± 3.575. This parallel supports the reliability and reproducibility of the measurements across different populations. Pearson correlation coefficients were utilized to assess the strength and direction of associations between the ANB angle and four alternative cephalometric parameters. These coefficients range from -1 to +1, with values approaching either extreme indicating stronger relationship. Among the variables studied, the Yen angle exhibited the strongest negative correlation with the ANB angle (r = -0.70), suggesting an inverse relationship: as the Yen angle increases, the ANB angle tends to decrease. Similar observations have been made by Kapadia *et al.* [12] and Soni *et al.* [11], who also reported a strong inverse relationship between these two metrics. The Beta angle (r = -0.66) also showed a robust negative correlation with ANB, aligning closely with the findings of Mittal *et al.* [14], who reported a negative correlation coefficient of -0.65. The K angle demonstrated a slightly weaker, yet still statistically significant, negative correlation (r = -0.61). In contrast, the Wits appraisal had a moderate positive correlation (r = 0.55) with the ANB angle, reflecting its reliance on dental rather than skeletal landmarks. This finding is consistent with the work of Gupta *et al.* [15], who similarly found a moderately positive association between ANB and Wits values. To better understand the predictive strength of these associations, the coefficient of determination (R^2^) was calculated. R^2^ indicated the proportion of variance in the ANB angle that each alternative parameter could explain. The Yen angle had the highest R^2^ value at 0.54, indicating it could explain 54% of the variability in ANB readings. The Beta angle followed with an R^2^ of 0.52, while the K angle explained 44% (R^2^ = 0.44). The Wits appraisal showed the lowest explanatory power, with an R^2^ of 0.37. These trends are consistent with prior studies by Kapadia *et al.* [12] and Mittal *et al.* [14], both of which reported superior R^2^ values for Yen and Beta angles compared to Wits. These findings support the clinical value of using Yen and Beta angles as viable alternatives to the ANB angle. Additional support for these findings can be found in previous research. Kapadia *et al.* [12] confirmed the strong association between the Beta and ANB angles. Baik *et al.* took a different approach by investigating the clinical utility of the Beta and Yen angles. They demonstrated that these angles, particularly when used in combination, provide a more nuanced understanding of sagittal jaw relationships. Their study emphasized that the Beta angle, with its strong correlation with the ANB angle, can serve as an effective alternative for assessing sagittal discrepancies, particularly in patients where variations in cranial base length may influence ANB readings. The Yen angle, as Baik *et al.* pointed out, provides a stable and reliable measure of the sagittal skeletal relationship due to its minimal sensitivity to cranial base alterations [16]. They concluded that combining both the Beta and Yen angles with traditional methods, such as the ANB, could significantly improve the accuracy of diagnosing sagittal discrepancies, leading to more precise treatment strategies. Ajins *et al.* [17] focused on the K angle and its relationship to the ANB angle, noting that the K angle provides a slightly weaker but still statistically significant correlation with the ANB. Their study illustrated that the K angle, although not as widely recognized as the Yen or Beta angles, holds value in clinical settings, particularly in cases where other parameters are difficult to measure due to patient positioning or technical challenges in cephalometric analysis. Ajins *et al.* also pointed out that the K angle may be beneficial in populations with certain anatomical variations, where other angles may show reduced reliability.

## Conclusion:

This study concludes that among the evaluated cephalometric indicators, the Yen angle emerged as the most reliable substitute for the ANB angle, followed by the Beta angle. The K angle demonstrated moderate reliability, while Wits appraisal showed weaker diagnostic consistency. Overall, the Yen and Beta angles offer strong predictive accuracy for sagittal skeletal assessment.

## References

[1] Bruks A (1999). Swed Dent J..

[2] Ghodasra R, Brizuela M (2023). Orthodontics, Cephalometric Analysis..

[3] Zere E (2018). Clin Cosmet Investig Dent..

[4] Kotula J (2022). Diagnostics (Basel)..

[5] Boccalari E (2025). J Clin Med..

[6] Albarakati S.F (2012). Dentomaxillofac Radiol..

[7] Nagar S (2014). Contemp Clin Dent..

[8] Mohelay N (2024). Cureus..

[9] Singh G (2016). J Clin Diagn Res..

[10] Ahmed M (2018). Dental Press J Orthod..

[11] Soni G (2021). European Journal of Molecular & Clinical Medicine..

[12] Kapadia R.M (2017). International Journal of Clinical Dentistry and Research..

[13] Neela PK (2009). World Journal of Orthodontics..

[14] Mittal A (2016). J Indian Orthod Soc..

[15] Gupta PK (2016). J Contemp Dent..

[16] Baik CY (2004). Am J Orthod Dentofacial Orthop..

[17] Ajins CB (2024). International Journal of Orthodontic Rehabilitation..

